# Intermittent Fasting Attenuates Hallmark Vascular and Neuronal Pathologies in a Mouse Model of Vascular Cognitive Impairment

**DOI:** 10.7150/ijbs.75188

**Published:** 2022-10-17

**Authors:** Vismitha Rajeev, David Y. Fann, Quynh Nhu Dinh, Hyun Ah Kim, T. Michael De Silva, Dong-Gyu Jo, Grant R. Drummond, Christopher G. Sobey, Mitchell K.P. Lai, Christopher Li-Hsian Chen, Thiruma V. Arumugam

**Affiliations:** 1 Memory Aging and Cognition Centre, Department of Pharmacology, Yong Loo Lin School of Medicine, National University of Singapore, Singapore; 2 Department of Biochemistry, Yong Loo Lin School of Medicine, National University of Singapore, Singapore; 3 Healthy Longevity Translational Research Program, Yong Loo Lin School of Medicine, National University of Singapore, Singapore; 4 Centre for Healthy Longevity, National University Health System (NUHS), Singapore; 5 Centre for Cardiovascular Biology and Disease Research, Department of Microbiology, Anatomy, Physiology and Pharmacology, School of Agriculture, Biomedicine and Environment, La Trobe University, Bundoora, VIC, Australia; 6 School of Pharmacy, Sungkyunkwan University, Suwon, Republic of Korea

**Keywords:** Vascular dementia, Vascular cognitive impairment, Intermittent fasting, Neuronal death, White matter lesions, Blood brain barrier breakdown, Chronic cerebral hypoperfusion

## Abstract

**Background -** Chronic cerebral hypoperfusion (CCH) is an important pathophysiological mechanism of vascular cognitive impairment (VCI). The heterogeneous effects of CCH complicate establishing single target therapies against VCI and its more severe form, vascular dementia (VaD). Intermittent fasting (IF) has multiple targets and is neuroprotective across a range of disease conditions including stroke, but its effects against CCH-induced neurovascular pathologies remain to be elucidated. We therefore assessed the effect of IF against CCH-associated neurovascular pathologies and investigated its underlying mechanisms.

**Methods -** Male C57BL/6NTac mice were subjected to either ad libitum feeding (AL) or IF (16 hours of fasting per day) for 4 months. In both groups, CCH was experimentally induced by the bilateral common carotid artery stenosis (BCAS) method. Sham operated groups were used as controls. Measures of leaky microvessels, blood-brain barrier (BBB) permeability, protein expression of tight junctions, extracellular matrix components and white matter changes were determined to investigate the effect of IF against CCH-induced neurovascular pathologies.

**Results -** IF alleviated CCH-induced neurovascular pathologies by reducing the number of leaky microvessels, BBB breakdown and loss of tight junctional proteins. In addition, IF mitigated the severity of white matter lesions, and maintained myelin basic protein levels, while concurrently reducing hippocampal neuronal cell death. Furthermore, IF reduced the CCH-induced increase in levels of matrix metalloproteinase (MMP)-2 and its upstream activator MT1-MMP, which are involved in the breakdown of the extracellular matrix that is a core component of the BBB. Additionally, we observed that IF reduced CCH-induced increase in the oxidative stress marker malondialdehyde, and increased antioxidant markers glutathione and superoxide dismutase. Overall, our data suggest that IF attenuates neurovascular damage, metalloproteinase and oxidative stress-associated pathways, and cell death in the brain following CCH in a mouse model of VCI.

**Conclusion -** Although IF has yet to be assessed in human patients with VaD, our data suggest that IF may be an effective means of preventing the onset or suppressing the development of neurovascular pathologies in VCI and VaD.

## Introduction

Vascular cognitive impairment (VCI) embodies a spectrum of cognitive deficits that range from mild cognitive impairment to vascular dementia (VaD). Due to a considerable increase in the aging population, VCI is becoming a major public health concern worldwide 1,2. VCI is associated with cerebrovascular diseases that arise from vascular pathological processes such as atherosclerosis, microvascular protein deposits, haemorrhages and microbleeds 3-8. These vascular pathologies lead to a state of reduced blood flow to the brain that is referred to as chronic cerebral hypoperfusion (CCH) 9-13. Decreased cerebral perfusion has been reported to correlate with dementia severity, and has shown to be a predictive marker to identify individuals with mild cognitive impairment who develop dementia 14,15. VaD patients have neurovascular pathologies such as blood-brain barrier (BBB) dysfunction, vascular damage, white matter lesion (WML) formation, glial activation, neuronal loss, and hippocampal atrophy 16-18. The bilateral common carotid artery stenosis (BCAS) mouse model of VCI is based on inducing brain CCH, and is a well-established model that mimics the neurovascular pathology observed in VaD patients 19,20.

CCH induces a cascade of cellular and molecular mechanisms that contributes to the pathogenesis of VCI - including oxidative stress and inflammation. CCH has been reported to increase the levels of matrix metalloproteinases (MMPs), proinflammatory cytokines such as interleukin-1 beta (IL-1β), interleukin-6 (IL-6) and tumour necrosis factor (TNF) 17,21-26, and promote cortical microbleeds, which are structural lesions in the brain that compromise cerebrovascular integrity and perfusion 27. A high frequency of microbleeds have been associated with an increased risk of cognitive deterioration and dementia 28-30. While microbleeds in the brain account for gross pathology in CCH, at the cellular level, the structural and functional integrity of the brain depends on the delivery of substrates between the blood and the brain through the blood brain barrier (BBB). Indeed, it has recently been reported that BBB dysfunction may be an initiator of WMLs and cognitive decline in VaD 31-33.

Intermittent fasting (IF) is defined as an eating pattern that cycles between periods of eating and fasting. IF has been extensively reported to extend both health and lifespan, and decrease the development of age-related disorders including cardiovascular, metabolic and neurodegenerative diseases 34. Recently, IF has gained much interest as being more effective than caloric restriction for inducing neuroprotective effects in the brain 35,36. Mechanistically, IF has been reported to enhance neuroprotection through the upregulation of neuroprotective proteins while reducing the activation of pathological pathways involving cellular stress, inflammasomes, and programmed cell death under ischemic conditions 37-42. However, the effects of IF on neurovascular pathology during CCH have not been investigated.

In this study, we demonstrate for the first time that IF promotes neuroprotective effects in a BCAS model of VaD by maintaining the integrity of the neurovascular structures in the brain. We specifically show that IF attenuated vascular pathology by reducing microvascular leakage and BBB dysfunction, while maintaining the expression of tight junction (TJ) proteins. IF was also effective in decreasing WML formation, hippocampal neuronal cell death and cell death markers, while maintaining myelin basic protein levels. Our data suggest that the effects of IF on the structural integrity of the neurovasculature may be mediated through mechanisms that decrease oxidative stress and matrix metalloproteinase expression. Overall, our findings indicate that prophylactic IF may be a potential therapy in reducing and preventing neurovascular pathology associated with VaD.

## Materials and Methods

### Experimental Animals

**Figure 1:**
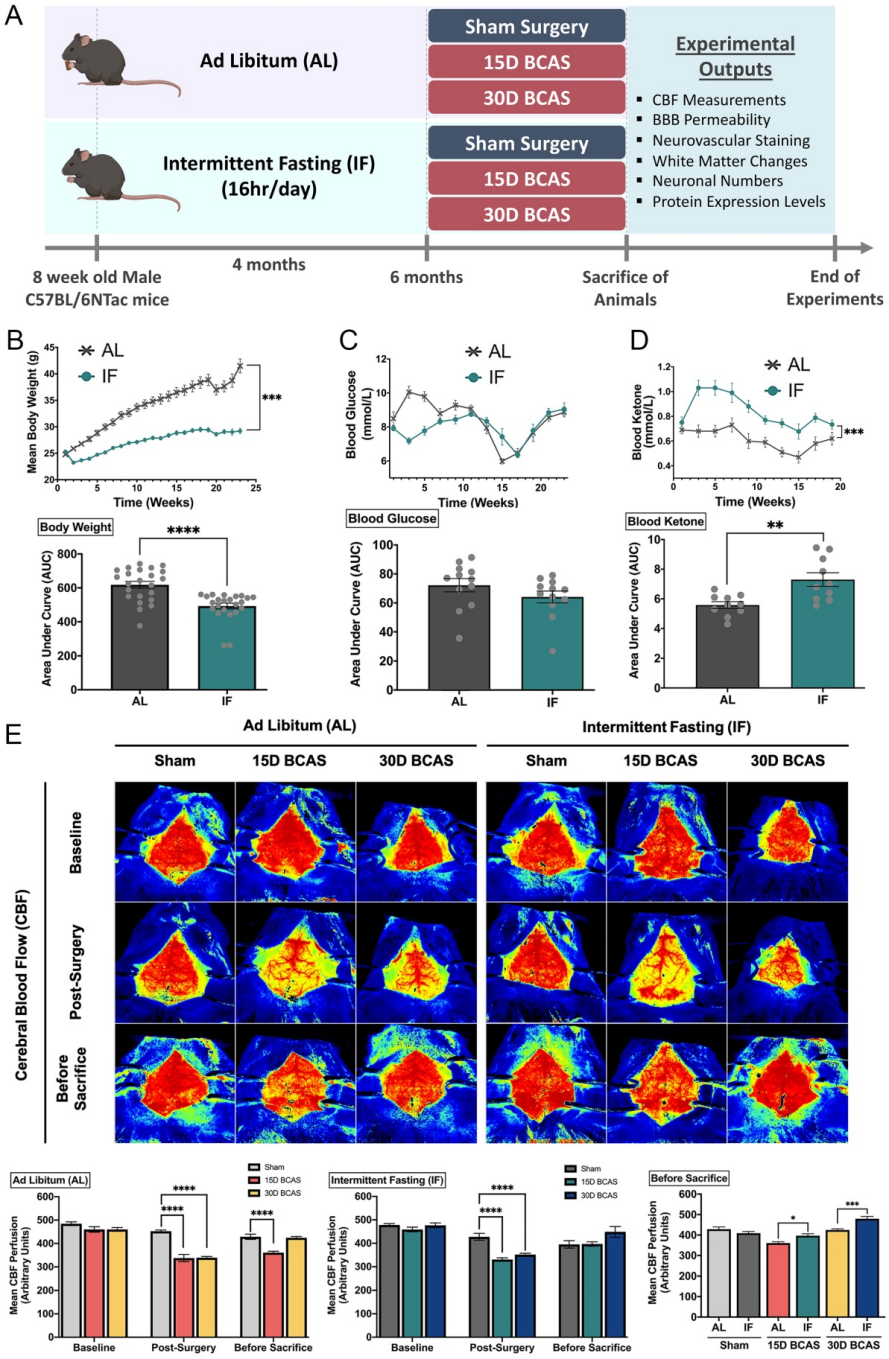
** Effect of intermittent fasting on physiological measurements and blood flow in the brain following BCAS in a mouse model of VCI. A.** Schematic representation of the experimental design and outputs performed. **B.** Mean body weight measurements over 25 weeks. ***P<0.001, ****P<0.0001 compared with AL. **C.** Blood glucose measurements over 25 weeks. **D.** Blood ketone measurements over 25 weeks. **P<0.01, ***P<0.001 compared with AL. **E.** Representative contrast images and quantification of CBF at baseline, effective blood flow reduction post-surgery, and the final level of cerebral blood flow before sacrifice at each individual end point demonstrated significant changes in cerebral blood flow following BCAS. Histographs illustrating the comparison of CBF levels at the sacrifice timepoint. The rate of blood flow was expressed in perfusion units (PU) using the PeriMed Software. *P<0.05, ***P<0.001 compared with corresponding AL BCAS; ****P<0.0001 compared with corresponding Sham. Data are represented as mean ± standard error of the mean of n=10-12 mice in each experimental group. Abbreviations: AL, ad libitum; BBB, blood-brain barrier; BCAS, bilateral common carotid artery stenosis; CBF, cerebral blood flow; IF, intermittent fasting; VCI, vascular cognitive impairment.

Six-week-old wild-type male C57BL/6NTac mice (n=150) were obtained from InVivos, Singapore and housed upon arrival at the National University of Singapore Animal Facility. As shown in **Figure 1A**, at eight weeks of age, the mice were randomly assigned to either the *ad libitum* (AL) or the intermittent fasting (IF) diet regimen. Mice under the IF regimen underwent 16 hours of fasting every 24-hour period, with food available for the remaining 8 hours from 07:00 to 15:00 for four months (Lights on at 7:00, lights off at 18:00). All in vivo experimental procedures were approved by the National University of Singapore, Singapore Animal Care and Use Committee and performed according to the guidelines set forth by the National Advisory Committee for Laboratory Animal Research, Singapore. All efforts were made to minimize any suffering and number of animals used. All sections of the manuscript were performed and reported in accordance to ARRIVE (Animal Research Reporting In Vivo Experiments) guidelines. In a separate set of experiments, animals from both AL and IF groups (10-20 animals per group) underwent physiological measurements such as body weight, blood glucose and ketone levels throughout the four months as shown in **Figure 1B-D**.

### Bilateral Common Carotid Artery Stenosis (BCAS) Mouse Model

At six months of age, AL and IF experimental animals were further divided into three experimental groups: Sham, 15-day bilateral common carotid artery stenosis (BCAS) and 30-day BCAS. C57BL/6NTac mice were subjected to chronic cerebral hypoperfusion injury by bilateral common carotid artery stenosis (BCAS) surgery, as previously described 20. Briefly, the animals were anesthetized with isoflurane (1.5- 2% at a flow rate of 0.4-0.8 litre/min), and a vertical midline incision was made in the neck. The left and right common carotid arteries were isolated from surrounding tissues and exposed individually, and loosely ligated with silk thread for easy manipulation of the arteries. The common carotid arteries were each constricted with microcoils of an internal diameter 0.18mm, specifically designed for the mice (microcoil specifications: piano wire with gold plating, piano wire diameter 0.08mm, coiling pitch 0.5mm, and total length of 2.5mm; Sawane Spring Co. Ltd, Japan). The silk threads were removed, and the site of surgery was closed. The mice were monitored post-surgery until they were awake. Sham animals were subjected to a midline cervical incision and their common carotid arteries were exposed, but no microcoils were inserted around the arteries. The animals received BCAS surgery during feeding hours in the daytime between 8am-12pm. All animals were euthanised at their respective endpoints after BCAS for subsequent experimental analysis.

### Cerebral Blood Flow Measurements

The Laser Speckle Contrast Imager (PSI system, Perimed Inc.) was used to obtain high-resolution cerebral blood flow (CBF) measurements before BCAS (baseline), immediately after BCAS surgery (post-BCAS) and finally at the end points of BCAS (before sacrifice). As shown in **Figure 1E**, the brain regions of interest (ROI) between the bregma and the lambda were selected to measure arbitrary units of CBF in the area between the cerebral hemispheres. Briefly, the fur on the head was removed by shaving and the skull was exposed via a midline skin incision. The skull was cleaned gently using a cotton applicator with 1×phosphate-buffered solution (PBS). Throughout the imaging of CBF, the skull was kept moist and in order to improve imaging resolution, a non-toxic silicon oil was applied on the skull. Body temperature was maintained at 37 ± 0.5ºC throughout the measurement periods. In the PeriCam PSI System, a CCD camera (2448×2048 pixels) that can take images in real-time was installed 10cm above the skull (speed 120 frames per second). Images were then acquired and analysed using a dedicated software package (PIMSoft, Perimed Inc.).

### Immunoblot Analysis

Animals were euthanized by inhaled carbon dioxide and the brains were harvested at their respective time points. Different brain regions (cortex, hippocampus, and cerebellum) were isolated immediately on ice and frozen in dry ice for future analysis (n=4-7 in each experimental group). Detailed immunoblot analysis procedures were performed as previously described 43. Briefly, brain tissues were homogenised in lysis buffer (Thermo Fisher Scientific, #78510) and combined with protease (Thermo Fisher Scientific, #78443) and phosphatase (Thermo Fisher Scientific, #78428) inhibitors to prevent proteolysis and dephosphorylation of proteins respectively during extraction, and then combined with 2x Laemelli buffer (Bio-Rad Laboratories, Inc., Hercules, CA, USA). Protein samples were then separated on 5-12.5% v/v sodium dodecyl sulfate (SDS) gel matrixes. Polyacrylamide gel electrophoresis of SDS-treated proteins were separated based on their sizes. The proteins on the gels were then transferred onto nitrocellulose membranes to allow for probing. The nitrocellulose membranes were incubated with the following primary antibodies: ZO-1 (Thermo Fisher, 61-7300, 1:1000), Occludin (Invitrogen, 711500, 1:1000), Claudin-5 (Thermo Fisher, 35-2500, 1:1000), JAM-A (Santa Cruz, sc25629, 1:1000), MBP (Cell Signaling, #78896S, 1:1000), Cleaved Caspase-3 (Cell Signaling, #9664, 1:1000), Total Caspase-3 (Cell Signaling, #9662, 1:1000), MMP2 (Cell Signaling, #87809S, 1:1000), MMP9 (Millipore, AB19016, 1:1000), MT1-MMP (Cell Signaling, #13130S, 1:1000), Malondialdehyde (Abcam, ab6463, 1:1000), Superoxide Dismutase (Abcam, ab13498, 1:1000), Glutathione (Abcam, ab19534, 1:1000), Vinculin (Cell Signaling, #13901, 1:1000) and β-actin (Sigma-Aldrich, A5441, 1:1000) overnight at 4°C with agitation. Following primary antibody incubation overnight, membranes were washed for 10 minutes thrice with 1×TBST before incubation with horseradish peroxidase (HRP)-conjugated secondary antibodies of dilution 1:1000 (Goat Anti-Rabbit - Cell Signaling Technology, Danvers, MA, USA; Goat Anti-Mouse - Sigma-Aldrich, St. Louis, MO, USA) for 1 hour at room temperature with agitation. Following secondary antibody incubation, membranes were washed for 10 minutes thrice with 1×TBST. Finally, the membranes were imaged using ChemiDocXRS+ imaging system (Bio-Rad Laboratories, Inc., Hercules, CA, USA) after the substrate, enhanced chemiluminescence (ECL), was added (Bio-Rad Laboratories, Inc., Hercules, CA, USA). ImageJ software (Version 1.46; National Institute of Health, Bethesda, MD, USA) was used to quantify proteins in relation to their corresponding housekeeping gene (β-actin/Vinculin).

### DiI Vasculature Staining

At the end of each timepoint, a subset of animals (5 animals per experimental group) were deeply anaesthetized with isoflurane, and perfusion through the heart was performed as described previously 44. Cardiac perfusion began with 25mL of chilled 1×PBS (pH 7.4) followed by perfusion of 10mL DiI solution (Sigma Aldrich, D-282, #42364, Invitrogen), and finally perfusion with 25mL of chilled fixative 4% paraformaldehyde. Once the perfusion was completed, the brains were harvested and placed in vials containing 4% paraformaldehyde solution for immersion-fixation overnight at 4ºC. The brains were then embedded in 5% agarose before sectioning by a vibratome (Leica VT1200) at a 100μm thickness and collected on slides. The tissue sections were viewed under a confocal microscope under a Texas Red Fluorescence Filter (TissueGnostics, TissueFAXS Slide Scanner). 100 coronal sections to represent the area between the bregma and lambda of each brain were examined under the confocal microscope. The representative image chosen were consistent at the 50^th^ percentile, to best represent the area closest to the bregma.

### Evans Blue Staining

At the end of each timepoint, a subset of animals (6-7 animals per experimental group) underwent Evans Blue (EB) Staining analysis as described previously 45. Briefly, the animals were anesthetised using isoflurane. 2% EB solution (Sigma Aldrich, E-2129-10G, diluted in 1xPBS and filtered, 2ml/kg) was injected through the femoral vein into the mice. The EB was allowed to circulate in the mice for 30mins. At the end of the 30mins, the mice were euthanized with carbon dioxide. Cardiac perfusion of 20ml 1×PBS was performed to remove residual EB in the blood, after which the brain was harvested and stored in dry ice. Brain tissues were weighed, and the amount of 50% trichloroacetic acid (TCA) solution (diluted in 0.9% saline) was calculated based on a 1:2 ratio of weight (mg): volume (μl). The tissue was homogenised in TCA solution. The mixture was then sonicated (10 cycles, 30 seconds on, 30 seconds off). Subsequently, the mixture was then allowed to incubate overnight at 4ºC on a rotator to allow for the complete extraction of EB from the brain tissues. The TCA-lysate mixture was then centrifuged (30mins, 15,000rcf, 4ºC) and the supernatant was collected. The supernatant was loaded in a 96-well plate with supplementation of 95% ethanol in a 1:3 ratio, respectively. The plate was then placed into a spectrophotometer at 620nm to determine the EB concentration.

### Luxol Fast Blue and Cresyl Violet Staining

Luxol fast blue (LFB) staining and expression of myelin basic protein (MBP) revealed myelin integrity, while cresyl violet staining and expression of cell death markers were indicative assessments for neuronal loss in the hippocampus and different brain regions. Mouse brain tissues were fixed in 10% neutral buffered formalin and then processed into paraffin wax blocks. Coronal sections (3μm thick) were obtained via microtome sectioning (Leica Biosystems, RM2255) and collected on slides (Trajan Scientific and Medical, Australia). Luxol Fast Blue (LFB) staining was performed to detect the severity of white matter lesions (WMLs) in 5 regions (corpus collosum paramedian, corpus callosum median, caudoputamen, internal capsule and optic tract) in the brain. Briefly, tissue sections were de-waxed and rehydrated before immersion into the LFB solution (Abcam, UK) at 37ºC overnight. Excess staining was removed using 95% ethanol treatment followed by washing in deionized water. Gray and white matter differentiation was initiated with the treatment of 0.05% aqueous lithium carbonate (Abcam, UK) for 20s, followed by 70% ethanol until the nuclei was decolorized. Sections were then immersed in Cresyl Violet solution (Abcam, UK) for 5 mins and the excess staining was washed in deionized water. The sections were then dehydrated in an ethanol gradient (70-100%), and finally cleared in xylene and mounted onto the slides (Fluoromount-G Mounting Medium, Invitrogen). Brightfield images were taken under ×60 magnification using an Olympus upright Fluorescence Microscope BX53. A WML severity index was calculated by giving each brain region a grade of either: Grade 0 (normal), Grade 1 (disarrangement of nerve fibres), Grade 2 (formation of marked vacuoles), or Grade 3 (disappearance of myelinated fibres). The severity of the WMLs was scored by three blinded examiners and the number of neurons in CA1, CA2 and CA3 regions of the hippocampus were counted as previously described 46.

### Statistical Analysis

Experimental data were analysed by GraphPad Prism 8.0 software (GraphPad Software, San Diego, CA, USA). All values were expressed as mean ± standard error of the mean. One-way analysis of variance was used, followed by a Bonferroni post-hoc test to determine the significance between experimental groups. A P-value < 0.05 was deemed to be statistically significant. All experimental groups were compared against AL Sham conditions.

## Results

### Intermittent fasting reduces body weight and increases plasma ketone levels, while improving cerebral blood flow recovery following chronic cerebral hypoperfusion

The summarized study design includes the timing of interventions, and blood and tissue collection (**Fig. 1**). At eight weeks of age, the mice were randomly assigned to either the ad libitum (AL) or the intermittent fasting (IF) diet regimen (**Fig.1A**). Male C57BL/6NTac mice were fed a normal chow diet (on a caloric basis: 58% carbohydrate, 24% protein, and 18% fat). Mice were randomly assigned to Sham, 15-day BCAS and 30-day BCAS groups at six months of age when surgeries were performed. First, we monitored the physiological effects of IF on these mice. The IF group had a significantly lower body weight, a non-significant reduction in blood glucose level and a significant increase in blood ketone level than AL mice (**Fig. 1B-D**). We examined dynamic changes and recovery of cerebral blood flow (CBF) in the animals (**Fig. 1E**) using the Laser Speckle Imaging Technique. The CBF was measured at three time points i.e. baseline, post-surgery and before sacrifice in both AL and IF animals over 15 and 30 days following BCAS. At baseline, animals from all experimental groups had high CBF as indicated by the red perfusion colour. Sham did not show any reduction in CBF, as no microcoils were inserted onto the common carotid arteries. However, expectedly, following BCAS surgery (Post-surgery), AL and IF groups had a significant reduction in CBF compared to their corresponding Sham counterparts. Before sacrifice, at 15 days and 30 days post-surgery, CBF was found to increase and be restored in BCAS animals. Interestingly, for the first time in the VaD model, we show that IF was able to demonstrate a better CBF recovery as compared to AL following hypoperfusive injury.

### Intermittent fasting decreases microvascular leakage and maintains blood-brain barrier integrity following chronic cerebral hypoperfusion

**Figure 2:**
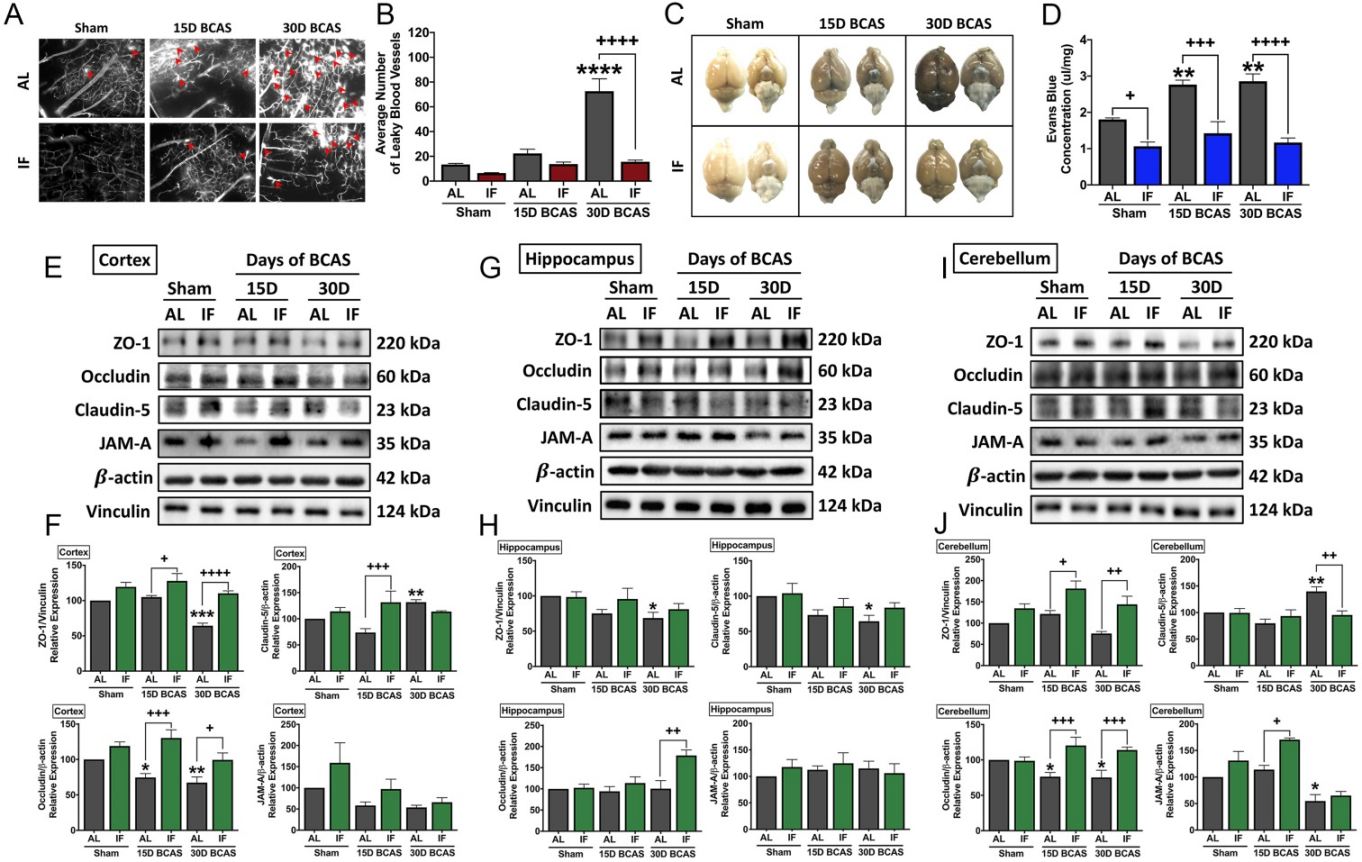
** Effect of intermittent fasting on leaky blood vessel incidence and blood-brain barrier integrity following BCAS. A-B.** Representative DiI staining images and quantification illustrating leaky blood vessel incidence in AL and IF mice following BCAS. Arrowheads point to leaky areas of the vessels. Data are represented as mean ± standard error of the mean of n=5 mice in each experimental group. ****P<0.0001 compared with AL Sham; ++++P<0.0001 compared with AL BCAS. **C-D**. Representative dorsal and ventral views of mouse brains injected with Evans Blue dye and respective quantification. Data are represented as mean ± standard error of the mean of n=5-7 mice in each experimental group. **P<0.01 compared with AL Sham; ^+^P<0.05, ^+++^P<0.001, ^++++^P<0.0001 compared to corresponding AL group. **E-J.** Representative immunoblots and quantification of tight junction proteins zonula occludens (ZO)-1, occludin, claudin-5 and junctional adhesion molecule (JAM)-A in the cortex (E-F), hippocampus (G-H) and cerebellum (I-J). Data are expressed as mean ± standard error of the mean. n=4-7 mice in each experimental group. β-actin or vinculin was used as a loading control. *P<0.05, **P<0.01, ***P<0.001 compared with AL Sham; ^+^P<0.05, ^++^P<0.01, ^+++^P<0.001, ^++++^P<0.0001, compared with corresponding AL BCAS. Abbreviations: AL, ad libitum; BCAS, bilateral common carotid artery stenosis; IF, intermittent fasting; VCI, vascular cognitive impairment.

We next analysed the effect of IF against hallmark vascular pathologies involving cerebrovascular structural and BBB integrities following CCH. The first vascular pathology examined was the number of microvascular leakages that occurred following CCH injury. Fluorescence imaging of vasculature staining using a carbocyanine dye perfusion indicated a significant increase in the average number of microvascular leaks at 30 days after BCAS in AL animals, while IF significantly reduced the number of microvascular leaks (**Fig. 2A-B**).

The second vascular pathology examined was the extent of BBB damage following CCH injury. We analysed BBB damage using the Evans Blue technique and additionally examined TJ protein expression levels via immunoblot analysis in the AL and IF mice following BCAS (**Fig. 2C-J**). BBB permeability after CCH was found to be significantly increased following 15 and 30 days of BCAS under AL conditions. In contrast, IF reduced BBB permeability in both sham and BCAS mice (**Fig. 2C-D**). To further analyse the structural integrity of the BBB, we next examined the expression levels of TJ proteins between endothelial cells in the cortex, hippocampus and cerebellar regions (**Fig. 2E-J**). There was evidence of reduced TJ expression following BCAS compared to Sham in AL animals, and IF was able to increase and maintain higher levels of TJ expression in all three brain regions. TJ protein zonula occludens (ZO-1) showed a significant reduction in expression levels following 30-day of BCAS at the cortex and the hippocampus, and a non-significant reduction in the cerebellum. Conversely, IF increased the expression of ZO-1 in the cortex and cerebellum at both 15-day and 30-day time points (**Fig. 2E-J**). Expression of occludin was significantly reduced following 15-day and 30-day BCAS at the cortex and cerebellum regions in AL animals, while IF increased occludin expression significantly at both 15-day and 30-day timepoints in the cortex and cerebellum, but only at the 30-day timepoint in the hippocampus (**Fig. 2E-J**). Claudin-5 levels were maintained following 15-day BCAS, but increased following 30-day BCAS in the cortex and cerebellum. In the hippocampus, Claudin-5 levels showed a significant reduction in AL mice following the 30-day BCAS timepoint. IF increased Claudin-5 levels in the cortex at the 15-day timepoint, and reduced Claudin-5 levels at the cerebellum at the 30-day timepoint. IF maintained Claudin-5 levels following 15-day and 30-day BCAS in the hippocampus (**Fig. 2E-J**). Junctional adhesion molecule (JAM-A) expression was reduced significantly in AL animals following BCAS in the cerebellum and non-significantly in the cortex, but maintained in the hippocampus. IF maintained JAM-A expression levels in the cortex and hippocampus, but increased expression at the 15-day timepoint in the cerebellum (**Fig. 2E-J**). These data thus indicated that IF likely protected against CCH-induced BBB breakdown by maintaining TJ proteins compared to the AL diet.

### Intermittent fasting attenuates white matter lesion injury, hippocampal neuronal loss and cell death following chronic cerebral hypoperfusion

**Figure 3:**
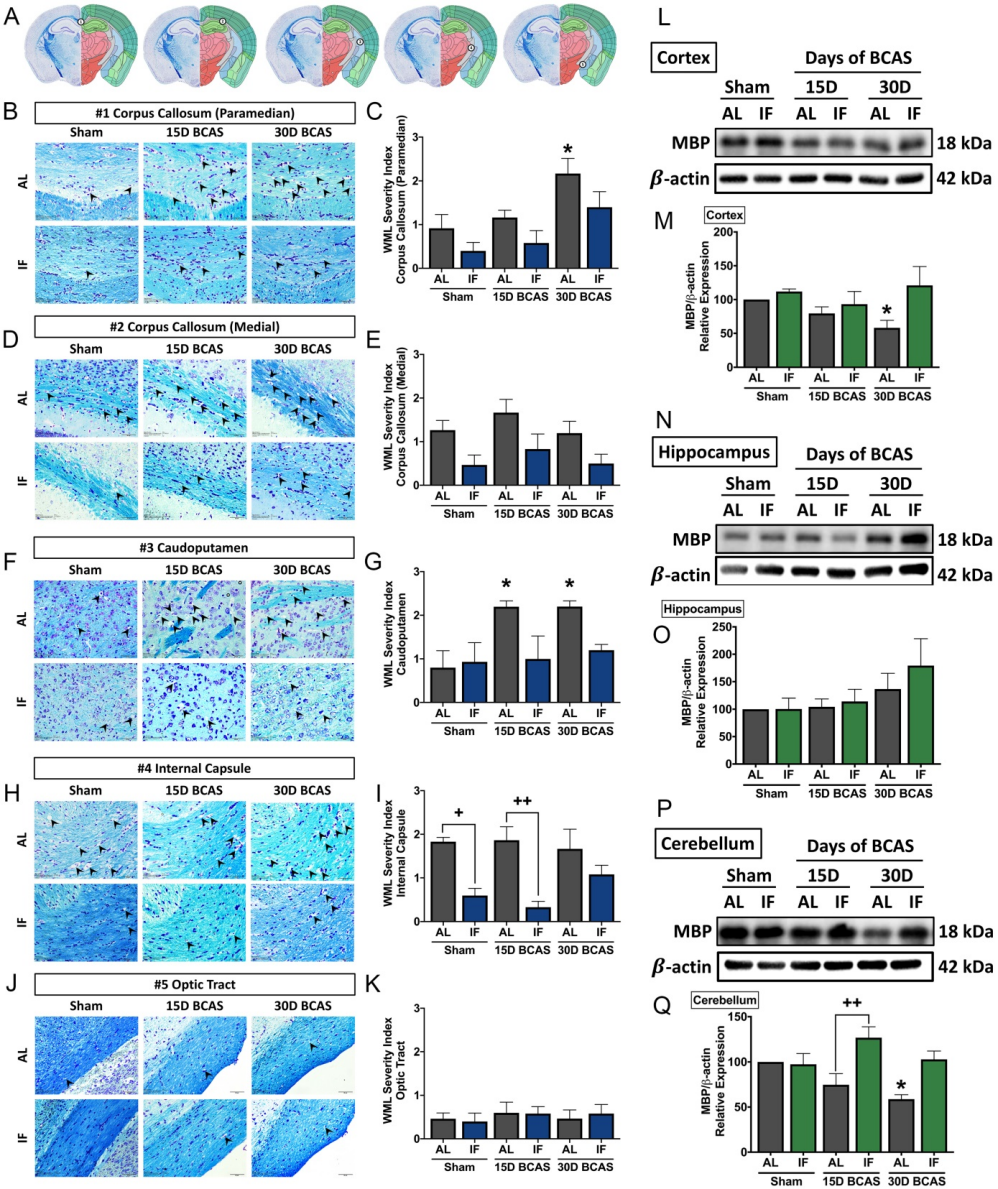
** Effect of intermittent fasting on white matter integrity in the brain following BCAS. A.** Schematic diagrams illustrating areas where white matter severity was measured, namely the corpus callosum (medial), corpus callosum (paramedian), caudoputamen, internal capsule and optic tract. **B-K.** Representative Luxol fast blue stained images and quantification illustrating white matter changes at the corpus callosum (paramedian) (B-C), corpus callosum (medial) (D-E), caudoputamen (F-G), internal capsule (H-I) and optic tract (J-K). The severity of white matter damage was graded as follows: Grade 0=no disruptions, Grade 1=disarrangement of nerve fibres, Grade 2=formation of marked vacuoles, and Grade 3=disappearance of myelinated fibres. Arrowheads point only to marked vacuoles throughout the image. Magnification x60. Scale bar, 20 μm. Images were taken under identical exposures and conditions. Data are represented as mean ± standard error of the mean. n=5 mice in each experimental group. *P<0.05 compared with AL Sham; ^+^P<0.05, ^++^P<0.01 compared with corresponding AL group. **L-Q.** Representative immunoblots and quantification of myelin basic protein (MBP) expression following BCAS in the cortex (L-M), hippocampus (N-O) and cerebellum (P-Q). Data are represented as mean ± standard error of the mean. n=4-7 mice in each experimental group. β-actin was used as a loading control. *P<0.05 compared with AL Sham; ^++^P<0.01 compared with corresponding AL BCAS. Abbreviations: AL, ad libitum; BCAS, bilateral common carotid artery stenosis; IF, intermittent fasting; VCI, vascular cognitive impairment.

We next analysed the effect of IF on neuronal pathologies such as white matter (WM) integrity, neuronal loss and cerebral cell death. The first neuronal pathology we examined was WM integrity and the corresponding WM protein known as myelin basic protein (MBP) (**Fig. 3**). WM integrity after 15 or 30 days of CCH was assessed in 5 brain regions including the corpus callosum (paramedian), corpus collosum (medial), caudoputamen, internal capsule and optic tract (**Fig. 3A-K**). The severity of WM damage was significantly increased in the corpus callosum (paramedian) at the 30-day timepoint (**Fig. 3B-C**) and caudoputamen at 15-day and 30-day timepoints (**Fig. 3F-G**) in AL mice following BCAS. At the corpus callosum (medial) (**Fig. 3D-E**) and internal capsule (**Fig. 3H-I**), AL animals showed a non-significant, but more severe disruption in white matter compared to their IF counterparts. We found no change in white matter disruption at the optic tract (**Fig. 3J-K**). IF was able to generally maintain a lower WM severity score as compared to their AL counterparts at all 5 regions, specifically significant at the internal capsule in Sham and 15-day timepoints. We found a significant decrease in the expression of MBP in the cortex and cerebellum at the 30-day timepoint in AL animals (**Fig. 3L-M, P-Q**). IF increased MBP expression in the cerebellum in BCAS mice at the 15-day timepoint (**Fig. 3P-Q**).

**Figure 4:**
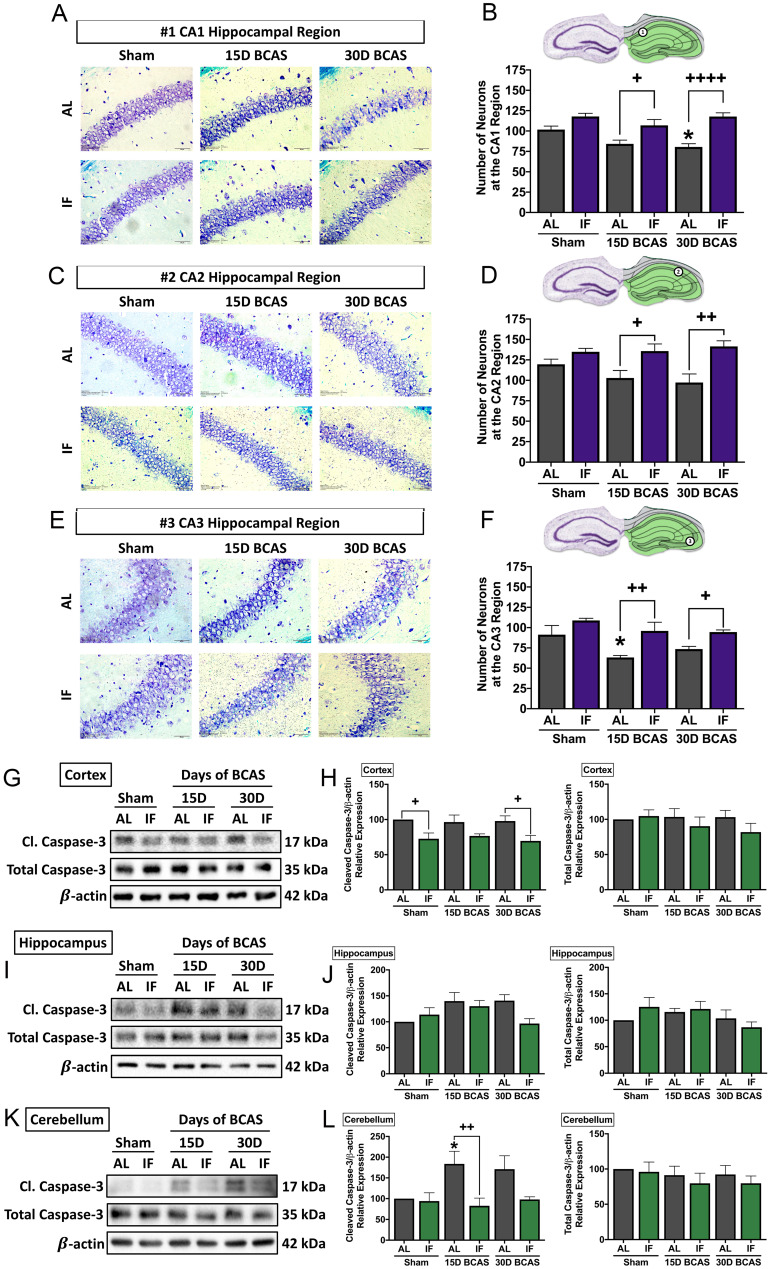
** Effect of intermittent fasting on hippocampal neuronal loss and apoptotic death in the brain following BCAS A-F.** Representative cresyl violet images and quantification illustrating Nissl positively stained neurons in hippocampal CA1 (A-B), CA2 (C-D) and CA3 (E-F) regions. Magnification x60. Scale bar, 20 μm. Images were taken under identical exposures and conditions. Data are represented as mean ± standard error of the mean. n=5 mice in each experimental group. *P<0.05 compared with AL Sham; ^+^P<0.05, ^++^P<0.01, ^++++^P<0.0001 compared with corresponding AL BCAS. **G-L**. Representative immunoblots and quantification illustrating apoptosis marker cleaved caspase-3 at the cortex (G-H), hippocampus (I-J) and cerebellum (K-L). Data are represented as mean ± standard error of the mean. n=4-7 mice in each experimental group. β-actin was used as a loading control. *P<0.05 compared with AL Sham; ^+^P<0.05, ^++^P<0.01 compared with corresponding AL group. Abbreviations: AL, ad libitum; BCAS, bilateral common carotid artery stenosis; Cl, cleaved; IF, intermittent fasting; VCI, vascular cognitive impairment.

The second neuronal pathology examined was hippocampal neuronal loss. BCAS-induced neuronal loss was evident in hippocampal regions (**Fig. 4A-F**). Sham controls in all three regions showed densely packed nuclei, but there were signs of neuronal loss at CA1, CA2 and CA3 regions evident following BCAS in AL animals. In the CA1 region, neuronal count was reduced at the 30-day timepoint in AL animals (**Fig. 4A-B**). In the CA2 region, the neuronal count showed a non-significant reduction in neuronal numbers following BCAS in AL animals (**Fig. 4C-D**). In the CA3 region, there was a reduction in neuronal numbers at the 15-day timepoint following BCAS in AL animals (**Fig. 4E-F**). IF was able to rescue degeneration of neurons significantly at the 15- and 30-day time points as compared to their AL counterparts in all three hippocampal regions (**Fig. 4A-F**).

The third and final neuronal pathology examined was cerebral cell death following CCH. Therefore, we analysed the expression levels of caspase-3 in the cortex, hippocampus and cerebellum as a marker for apoptotic cell death (**Fig. 4G-L**). Our data shows that although total caspase-3 levels remained unchanged in all three brain regions. Cleaved caspase-3 was found to show little change in the cortex (**Fig. 4G-H**), a non-significant increase in the hippocampus (**Fig. 4I-J**), and a significant increase at the 15-day timepoint at the cerebellum (**Fig. 4K-L**) in AL animals, which suggests CCH-mediated brain loss to be partly mediated by activation of the apoptotic caspase pathway. IF animals had reduced expression of cleaved caspase-3 at the sham and 30-day BCAS timepoints at the cortex, and at the 15-day timepoint at the cerebellum.

### Intermittent fasting decreases matrix metalloproteinase and oxidative stress levels in the brain following chronic cerebral hypoperfusion

**Figure 5:**
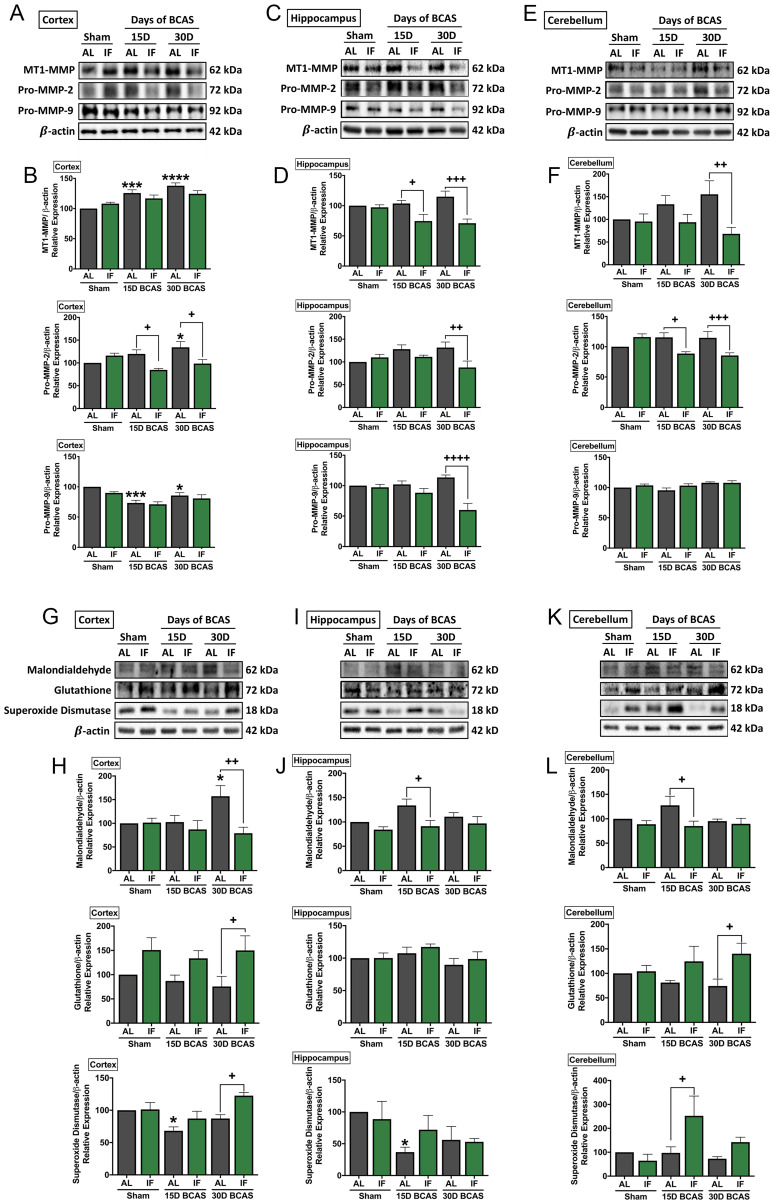
** Effect of intermittent fasting on matrix metalloproteinase and oxidative stress in the brain following BCAS. A-F.** Representative immunoblots and quantification illustrating matrix metalloproteinase (MMP)-2, its upstream membrane type (MT)-1 MMP (MT1MMP) and MMP-9 levels in the cortex (A-B), hippocampus (C-D) and cerebellum (E-F). Data are represented as mean ± standard error of the mean. n=5-7 mice in each experimental group. β-actin was used as a loading control. *P<0.05, ***P<0.001, ****P<0.0001 compared with AL Sham; ^+^P<0.05, ^++^P<0.01, ^+++^P<0.001, ^++++^P<0.0001 compared with corresponding AL BCAS. **G-L.** Representative immunoblots and quantification illustrating oxidative stress marker, malondialdehyde, and anti-oxidant markers, glutathione and superoxide dismutase in the cortex (G-H), hippocampus (I-J) and cerebellum (K-L). Data are expressed as mean ± standard error of the mean. n=4-7 mice in each experimental group. β-actin was used as a loading control. *P<0.05 compared with AL Sham; ^+^P<0.05, ^++^P<0.01 compared with corresponding AL BCAS. Abbreviations: AL, ad libitum; BCAS, bilateral common carotid artery stenosis; IF, intermittent fasting; VCI, vascular cognitive impairment.

As various cellular and molecular pathways have been implicated in CCH-induced brain injury, we postulated the involvement of matrix metalloproteinases (MMPs) in the breakdown of the extracellular matrix in the brain that could mediate both vascular and neuronal pathologies (**Fig. 5**). Our data established evidence for significantly increased expression of membrane type-1 matrix metalloproteinase (MT1-MMP) at the cortex, and non-significant increase in MT1-MMP levels in the hippocampus and cerebellum following BCAS (**Fig. 5A-F**). IF was able to significantly reduce MT1-MMP levels at the 15-and 30-day timepoints in the hippocampus and at the 30-day timepoint in the cerebellum (**Fig. 5C-F**). Downstream pro-MMP-2 showed a significant increase following BCAS in the cortex at the 30-day timepoint, and a non-significant increase in the hippocampus and cerebellum in AL animals (**Fig. 5A-F**). IF was able to significantly reduce the CCH-induced increase of pro-MMP-2 levels at the cortex and cerebellum at the 15-day and 30-day time points, and at 30-day timepoint in the hippocampus (**Fig. 5A-F**). Additionally, we found a decrease in pro-MMP-9 levels at the 15-day and 30-day timepoints in the cortex, but no changes in expression of pro-MMP-9 at the hippocampus and cerebellum, although pro-MMP-9 levels were downregulated in the hippocampus in IF BCAS animals at the 30-day timepoint (**Fig. 5A-F**) suggesting that the MMP-9 pathway is not induced during CCH-associated injury.

Another mechanism we postulated to be involved in CCH-induced brain injury was oxidative stress. We, therefore, analysed oxidative stress markers in the cortex, hippocampus and cerebellum (**Fig. 5G-L**). Our data showed that the oxidative stress marker, malondialdehyde, was significantly increased following 30 days of BCAS injury in AL animals at the cortex, but non-significantly in the hippocampus and cerebellum (**Fig. 5G-L**). IF reduced malondialdehyde levels at the 30-day timepoint in the cortex and at the 15-day timepoint in the hippocampus and cerebellum (**Fig. 5G-L**). Antioxidant glutathione levels were non-significantly lower in AL animals at the cortex and cerebellum following BCAS, and at the hippocampus, glutathione levels remained unchanged. IF was able to significantly increase expression levels of glutathione at the cortex and cerebellum at the 30-day timepoint, but showed no change at the hippocampus (**Fig. 5G-L**). Antioxidant superoxide dismutase was lower at the 15-day timepoint at the cortex and hippocampus but showed non-significant lower levels at the cerebellum. IF was able to increase superoxide dismutase levels at the cortex and cerebellum at the 30-day and 15-day time points respectively (**Fig. 5G-L**).

## Discussion

**Figure 6:**
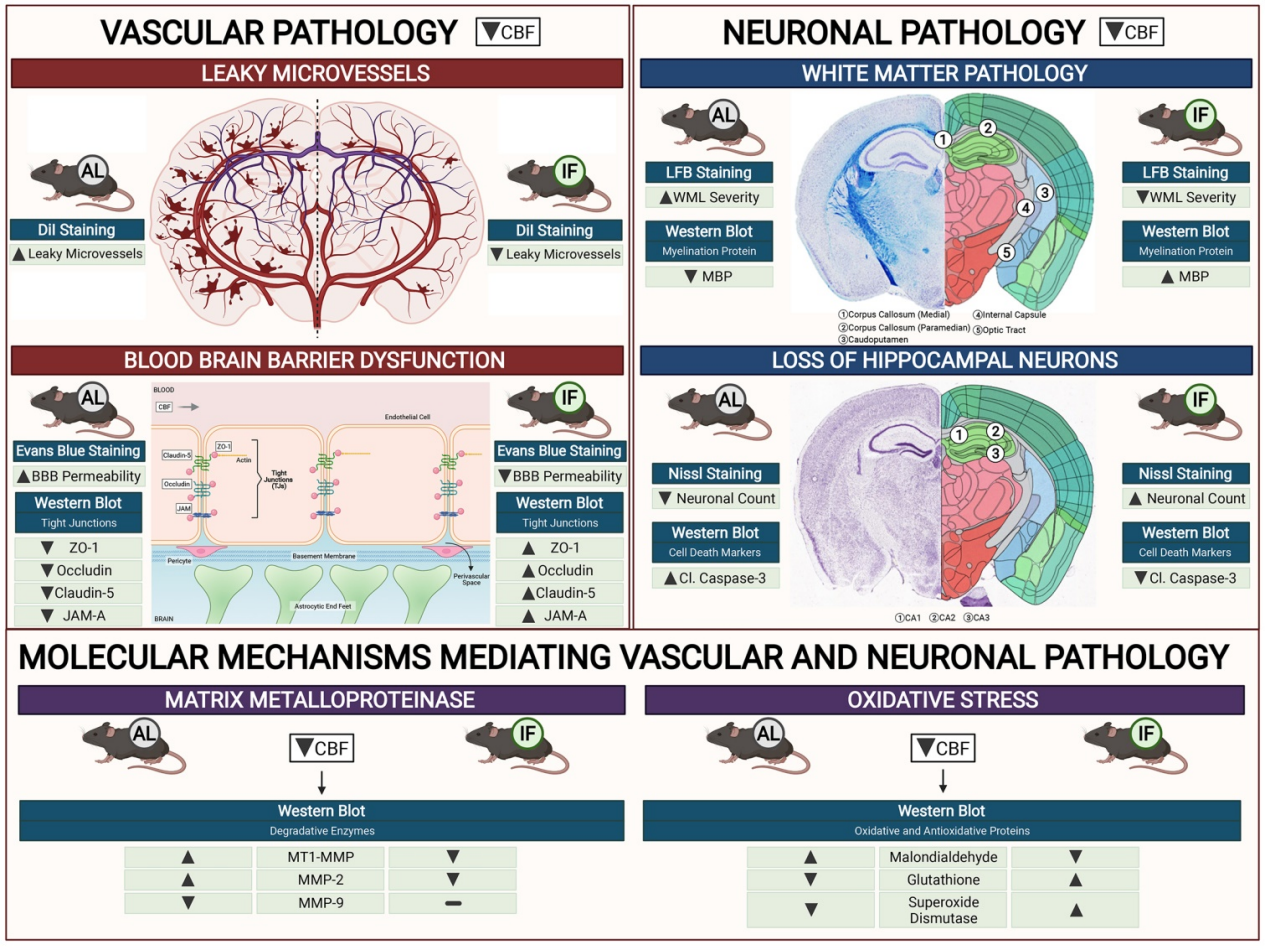
** Schematic diagram illustrating the effects of intermittent fasting on the molecular mechanisms mediating vascular and neuronal pathology in the brain following BCAS in a mouse model of VCI.** Vascular pathology in the brain following decreased cerebral blood flow (CBF) is defined as changes in the number of leaky microvessels and damage to the blood brain barrier (BBB). DiI staining was used to stain the vasculature, and IF was found to decrease the number of leaky microvessels in the brain following CCH. Evans blue staining was used to assess the extent of BBB damage in the brain. IF was found to decrease BBB permeability through increased tight junction proteins zonula-occludens (ZO)-1, Occludin, Claudin-5 and junctional adhesion molecule (JAM)-A. Vascular pathology in the brain following decreased CBF is defined as changes in the white matter pathology and loss of hippocampal neurons. Luxol fast blue (LFB) staining method was used to stain and image 5 different white matter regions (corpus callosum medial, corpus callosum paramedian, caudoputamen, internal capsule and optic tract). IF decreased the severity of white matter damage in the brain following CCH, through maintenance of myelin basic protein (MBP) levels. Nissl staining was used to visualise the hippocampal neurons in the brain, and IF was found to be able to increase neuronal counts through reducing apoptotic death indicated by the levels of cleaved caspase-3 in the brain. IF was found to reduce membrane-type 1 MMP (MT1-MMP) and MMP2 levels in the brain following CCH. However, MMP9 was not involved in breaking down the extracellular matrix following CCH. IF reduced oxidative stress as indicated through markers of lipid oxidation such as malondialdehyde, and antioxidative glutathione, and superoxide dismutase levels.

Our study establishes a consensus of the beneficial effect of IF against age-related diseases. The effect of IF achieved through intermittent metabolic switching which implies recurring periods of a bioenergetic challenge. IF leads to depletion of liver glycogen stores and production of ketones from adipose-cell-derived fatty acids, followed by recovery period. This metabolic switch results in coordinated molecular and cellular adaptations involving various signaling cascades. Current findings indicate such a metabolic switch protects against CCH-induced neurovascular pathology in an animal model of vascular dementia. Prophylactic IF attenuated microvascular leakage, BBB permeability, TJ breakdown, white matter injury, neuronal loss and cell death through a reduction in MMP and oxidative stress levels (**Fig. 6**). Collectively, these data are the first to demonstrate the effect of IF on vascular and neuronal pathologies following CCH injury, identifying IF as a potential therapeutic intervention for VCI.

Following CCH, several hemodynamic changes occur in response to decreased blood flow in the brain. BCAS surgery induces a reduction of global cerebral blood flow by approximately 30-40% before collateral development and recovery of blood flow 46. Microscopic imaging of capillaries in the brain have revealed local heterogeneities in cortical blood flow supply during hypoperfusion, such as reduced red blood cell perfusion 47. Our present data demonstrate that IF can alleviate vascular damage induced under hypoperfusion conditions, as IF animals had greater cerebral blood flow recovery at 15 and 30 days after BCAS surgery. In order to investigate the deep structural changes, we used a vasculature staining technique to observe vascular integrity. We showed that the number of microvascular leakages was lower in IF than AL animals during CCH. Leaky microvasculature are structural abnormalities on small vessels that lead to reduced cerebral perfusion, and are associated with aging and cognitive decline 48,49. Interestingly, we observed a rightward shift of leaky vessel formation in the brain at increasing time points. While we did not collect data between 15- and 30-days' time points to study the gradual changes of microvessel leakage, it may be attributed to repeated compromise of the blood vessels due to deteriorating brain pathology. Nevertheless, we are able to provide strong evidence that IF maintained vascular integrity, which improved CBF in the brain following hypoperfusion.

We investigated BBB permeability following CCH, and the effects of prophylactic IF on the integrity of the BBB in order to understand the mechanisms involved. Increased BBB permeability has been reported in animal models of CCH and VCI patients 50. However, there were no previous studies on the effect of IF on BBB permeability. We showed that prophylactic IF was able to maintain the integrity of the BBB under hypoperfused conditions via the Evans Blue technique. Interestingly, the integrity of the BBB was improved even under Sham conditions, that may posit the possibility that IF improves BBB integrity under normal physiological conditions. Investigation into the expression levels of molecular targets of BBB integrity, namely the TJ proteins, revealed that BBB integrity was maintained with IF following CCH via upregulation of TJ protein expression. TJ proteins such as zonula occludens (ZO-1), Occludin, Claudin-5 and junctional adhesion molecule (JAM-A) act as molecular gates between endothelial cells of the BBB 51. Our data are consistent with similar effects of IF on TJ proteins in the gut vasculature 52. While TJ protein expression levels were assessed, immunofluorescence studies to visualise the localisation of these TJ proteins may prove useful to be examined in future studies. Pericyte coverage and count, a property of the BBB that contributes to its integrity was not examined in this study, but has been reported to be decreased in the brains of BCAS mice 53. The effect of IF on pericyte coverage may prove to be useful in future studies on BBB integrity during CCH.

Following CCH, WMLs are reported to have a presumed vascular origin, and are associated with cognitive decline in the elderly 54. Reduced blood flow to the white matter regions have been shown to predict the clinical development of WMLs in the brain 55. At the tissue level, the breakdown of myelin sheaths causes myelin basic protein (MBP) network disassembly. A decrease in MBP expression with CCH therefore demonstrates structural disruption of the myelin sheath and hence formation of WMLs 56. We showed a striking difference between the severity of WMLs and MBP expression levels in animals under AL and IF diets. We observed that IF animals subjected to BCAS exhibited less white matter injury and maintenance of MBP levels compared to those on the AL diet. Similar results have been reported in multiple sclerosis mouse models where IF reduced demyelination in the spinal cord 57.

Another macroscopic neuronal pathologic lesion during CCH is neurodegeneration which involves the loss of neurons particularly at the hippocampus that is associated with memory, behavioural changes and cognition 58. Sub-regions of the hippocampus - CA1, CA2 and CA3 are responsible for memory coding and retrieval. Specifically, the CA1 region is involved in the formation, consolidation, and retrieval of hippocampal-dependent memories 59; the CA2 region is involved in the formation of social memory 60, and the CA3 region is involved in memory processes and neurodegeneration 61. Tightly orchestrated programmed cell death signalling events are activated in response to CCH, for which cell death markers such as caspase-3 play an important contributing role in apoptosis and hippocampal atrophy 56. We found that neuronal loss, and apoptotic cell death activation were present in AL animals following CCH, consistent with a previous study 56. In this study, we demonstrate less neuronal loss in the CA1, CA2 and CA3 hippocampal regions in IF mice following CCH. This implicates a mechanism through which IF may improve cognitive functions. Previous evidence suggests that the cerebellum is involved in cognition and behaviour of animals 62. Hence, due to the increased levels of cleaved caspase-3 observed in the cerebellum, it may also be closely linked to cognitive loss during CCH. We showed that IF attenuated cerebellar caspase-3 activation, which thus may mediate improved cognitive performance. Moreover, IF was also able to decrease cleaved caspase-3 levels in the cortex of Sham animals implying that it was able to suppress apoptotic cell death at basal conditions for reasons and mechanisms that remains to be fully elucidated.

It is important to elucidate the mechanism(s) at the molecular and cellular levels by which IF alleviates both vascular and neuronal pathologies following CCH. Here, we investigated pathways previously implicated in BBB breakdown, namely the activation of matrix metalloproteinases (MMPs) and oxidative stress. Previously, an increase in matrix metalloproteinases, particularly MMP-2 under CCH has been reported 63,64. In our study, we showed a similar finding that MMP-2 was upregulated during CCH, and IF reduced the expression of MMP-2 in the brain. While the expression levels were assessed, the enzymatic activity of the MMPs may prove useful to examine in future studies. Similarly, there is also evidence that increased oxidative stress levels occur in the brain following CCH. We observed that IF reduced CCH-induced increases in malondialdehyde levels, suggesting that reduced lipid peroxidation in the brain is a plausible mechanism through which IF attenuates neurovascular pathologies. Furthermore, IF increased anti-oxidant glutathione and superoxide dismutase levels in the brain following CCH. Our study suggests that IF exerts protective effects against vascular and neuronal pathologies through reduced levels of MMP and oxidative stress.

The precise mechanisms through which IF promotes VaD pathological tolerance is not yet completely deciphered. Nevertheless, future work in systematically studying these mechanisms and the molecular pathways are warranted. However, it was previously established that IF protects against age-related diseases such as cardiovascular diseases and neurodegeneration by targeting dysregulated energy mechanisms, oxidative stress, mitochondrial dysfunction, impaired lysosome and proteasome function, inflammation, cellular senescence and promoting the expression of genes by modulating epigenetic mechanisms that are essential for plasticity and regeneration 65,66,67,68. These pleiotropic adaptations of IF sets the stage for future work in the field of VaD.

In summary, we have provided substantial evidence that prophylactic IF is effective in reducing vascular and neuronal pathologies following CCH. While our study establishes IF as a potential therapeutic approach for attenuating VCI-related pathologies, further research is needed to test its clinical potential.

## Supplementary Material

Supplementary figures.

Supplementary data.
