# Supplementary material for: Intermittent Fasting Attenuates Hallmark Vascular and Neuronal Pathologies in a Mouse Model of Vascular Cognitive Impairment
Source: Int J Biol Sci. 2022 Oct 17;18(16):6052–67. doi: 10.7150/ijbs.75188 (PMC9682544; doi:10.7150/ijbs.75188)
Supplement: Supplementary file 1 — Supplementary figures. [file ijbsv18p6052s1.pdf]

Supplementary Document

Original Immunoblots

Figure 2E

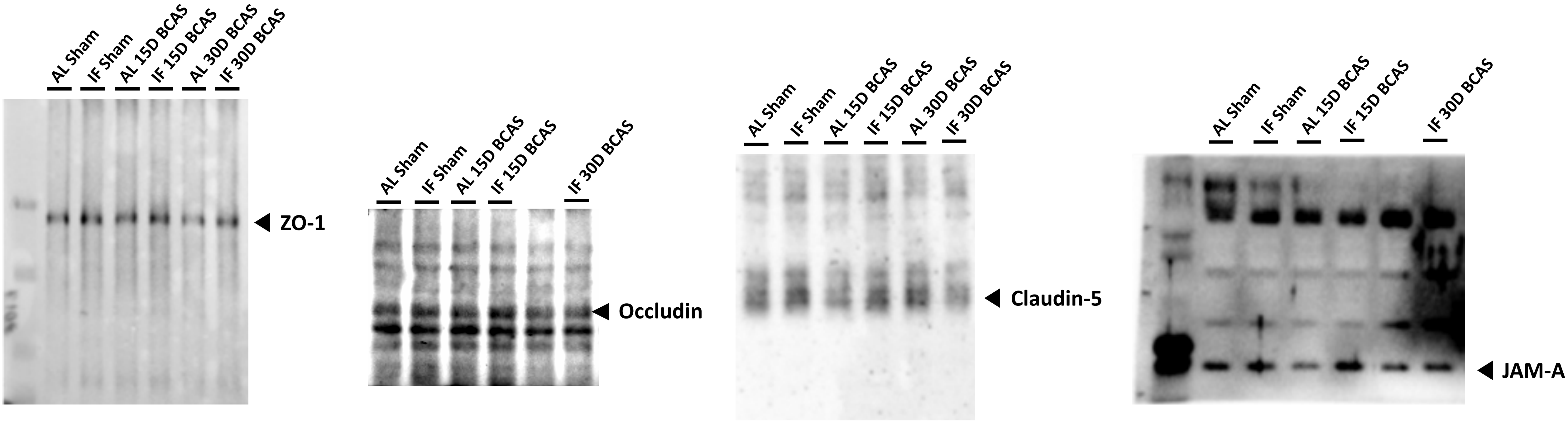

AL Sham
IF Sham
AL 15D BCAS
IF 15D BCAS
AL 30D BCAS
IF 30D BCAS
ZO-1
AL Sham
IF Sham
AL 15D BCAS
IF 15D BCAS
IF 30D BCAS
Occludin
AL Sham
IF Sham
AL 15D BCAS
IF 15D BCAS
AL 30D BCAS
IF 30D BCAS
Claudin-5
AL Sham
IF Sham
AL 15D BCAS
IF 15D BCAS
IF 30D BCAS
JAM-A

Figure 2G

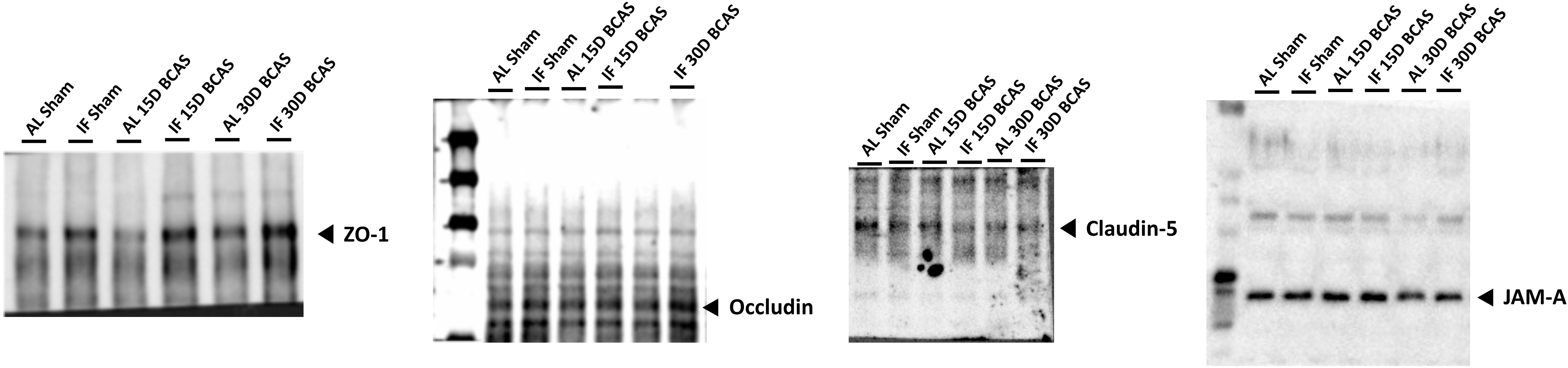
AL Sham
IF Sham
AL 15D BCAS
IF 15D BCAS
AL 30D BCAS
IF 30D BCAS
ZO-1
AL Sham
IF Sham
AL 15D BCAS
IF 15D BCAS
IF 30D BCAS
Occludin
AL Sham
IF Sham
AL 15D BCAS
IF 15D BCAS
AL 30D BCAS
IF 30D BCAS
Claudin-5
AL Sham
IF Sham
AL 15D BCAS
IF 15D BCAS
AL 30D BCAS
IF 30D BCAS
JAM-A

Figure 2I

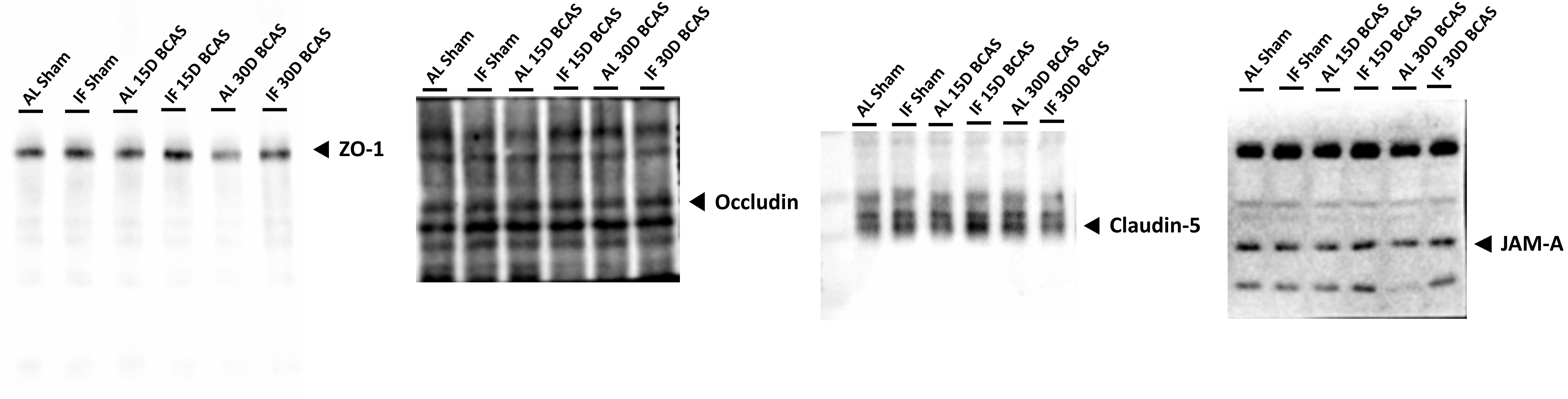
AL Sham
IF Sham
AL 15D BCAS
IF 15D BCAS
AL 30D BCAS
IF 30D BCAS
ZO-1
Occludin
Claudin-5
JAM-A

Figure 3L

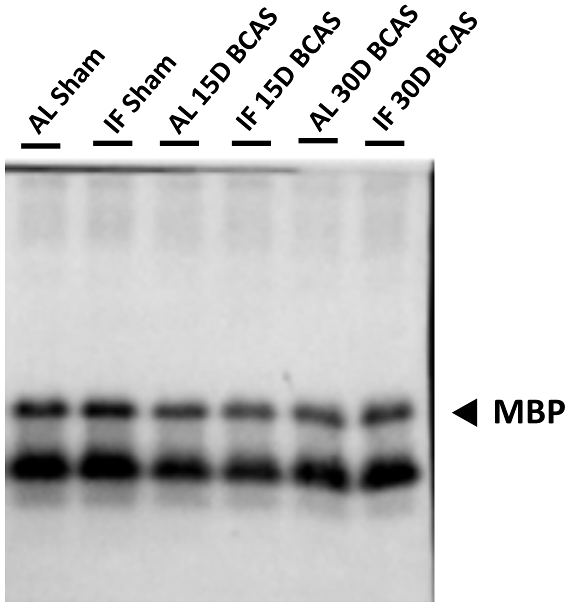
AL Sham
IF Sham
AL 15D BCAS
IF 15D BCAS
AL 30D BCAS
IF 30D BCAS
MBP

Figure 3N

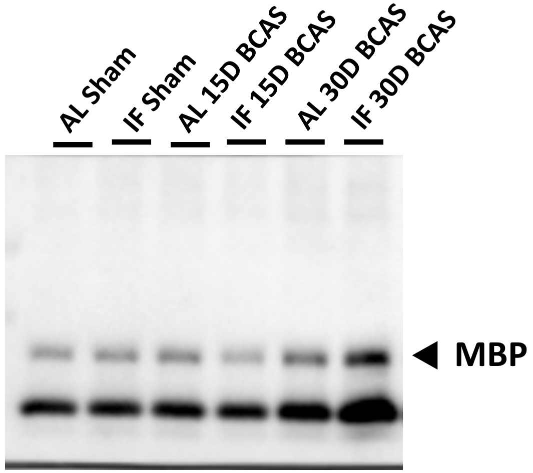
AL Sham
IF Sham
AL 15D BCAS
IF 15D BCAS
AL 30D BCAS
IF 30D BCAS
MBP

Figure 3P

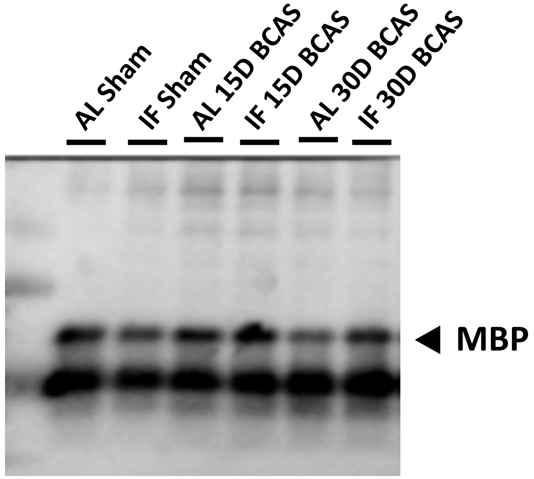
AL Sham
IF Sham
AL 15D BCAS
IF 15D BCAS
AL 30D BCAS
IF 30D BCAS
MBP

Figure 4G

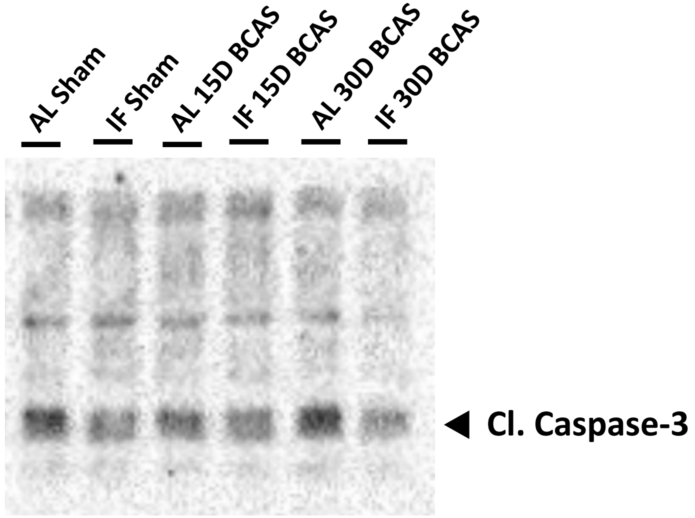
AL Sham
IF Sham
AL 15D BCAS
IF 15D BCAS
AL 30D BCAS
IF 30D BCAS
Cl. Caspase-3

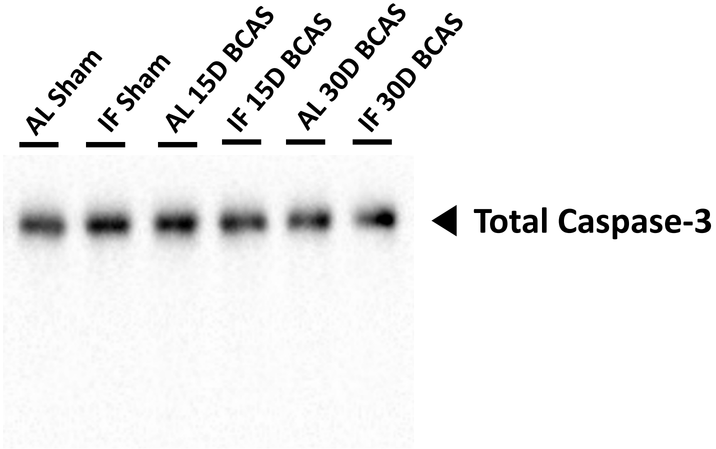
AL Sham
IF Sham
AL 15D BCAS
IF 15D BCAS
AL 30D BCAS
IF 30D BCAS
Total Caspase-3

Figure 4I

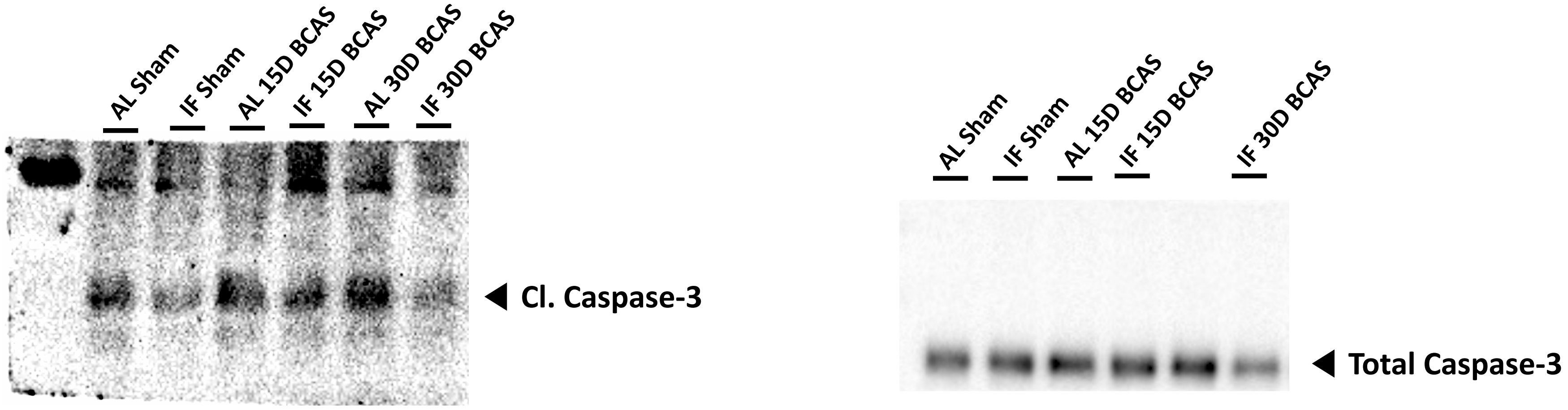
AL Sham
IF Sham
AL 15D BCAS
IF 15D BCAS
AL 30D BCAS
IF 30D BCAS
Cl. Caspase-3
AL Sham
IF Sham
AL 15D BCAS
IF 15D BCAS
IF 30D BCAS
Total Caspase-3

Figure 4K

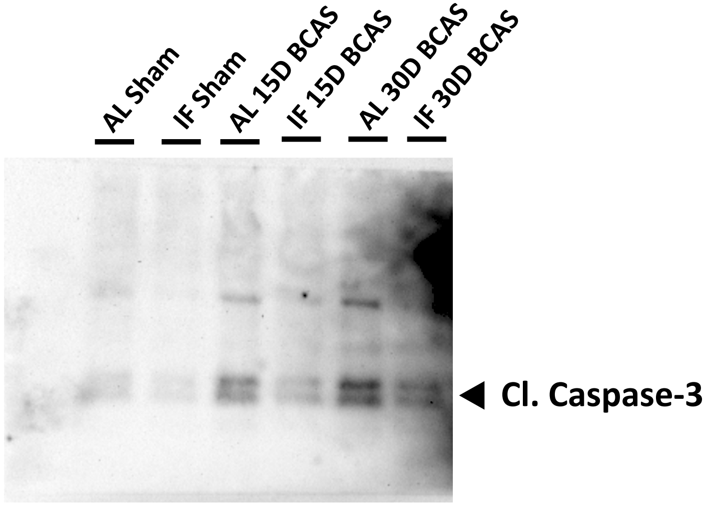
AL Sham
IF Sham
AL 15D BCAS
IF 15D BCAS
AL 30D BCAS
IF 30D BCAS
Cl. Caspase-3

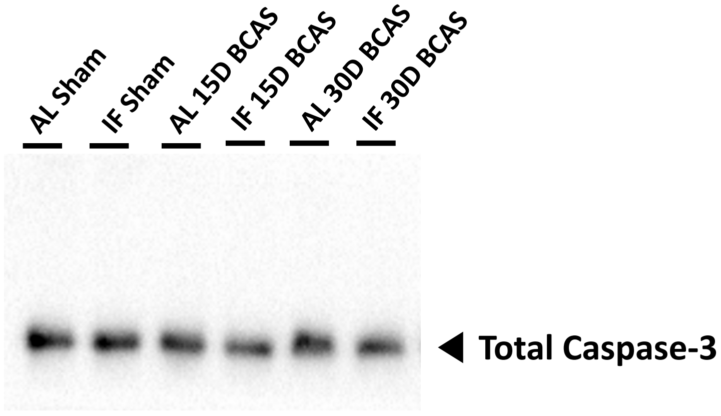
AL Sham
IF Sham
AL 15D BCAS
IF 15D BCAS
AL 30D BCAS
IF 30D BCAS
Total Caspase-3

Figure 5A

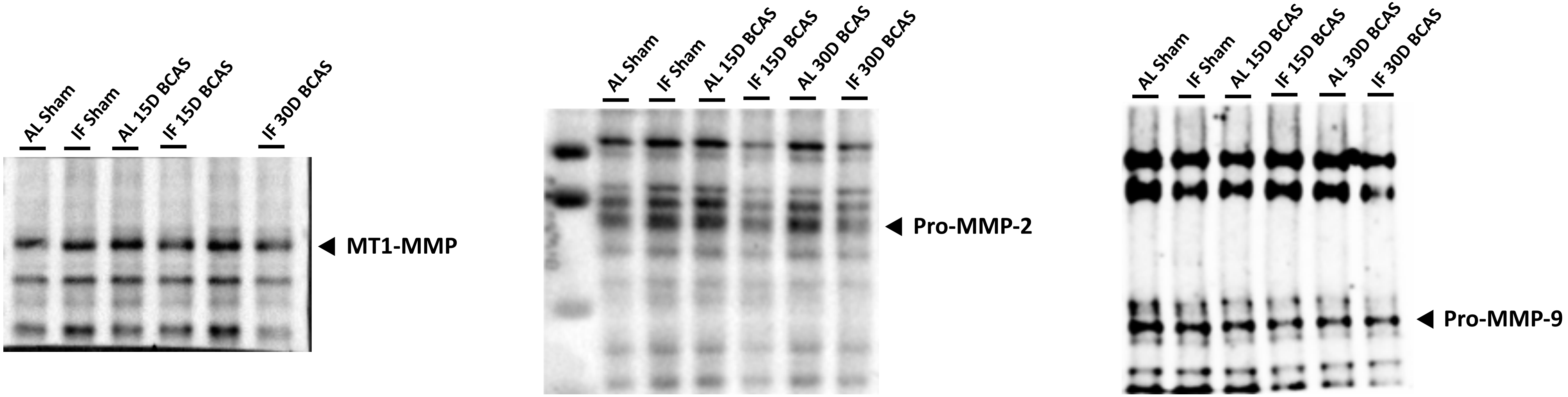

AL Sham
IF Sham
AL 15D BCAS
IF 15D BCAS
IF 30D BCAS
MT1-MMP
AL Sham
IF Sham
AL 15D BCAS
IF 15D BCAS
AL 30D BCAS
IF 30D BCAS
Pro-MMP-2
AL Sham
IF Sham
AL 15D BCAS
IF 15D BCAS
AL 30D BCAS
IF 30D BCAS
Pro-MMP-9

Figure 5C

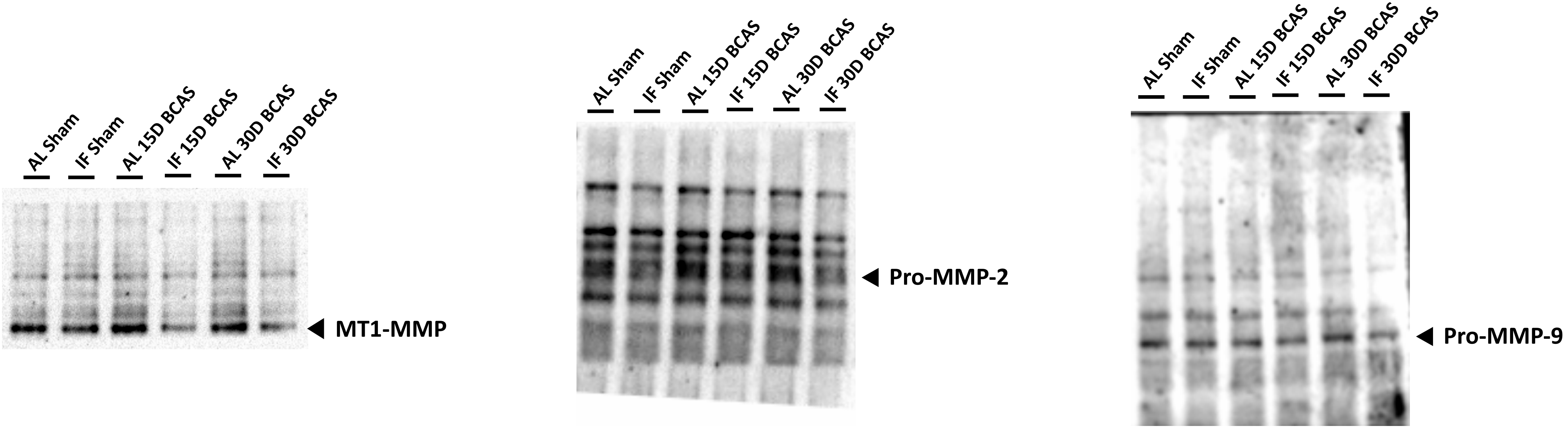
AL Sham
IF Sham
AL 15D BCAS
IF 15D BCAS
AL 30D BCAS
IF 30D BCAS
MT1-MMP
AL Sham
IF Sham
AL 15D BCAS
IF 15D BCAS
AL 30D BCAS
IF 30D BCAS
Pro-MMP-2
AL Sham
IF Sham
AL 15D BCAS
IF 15D BCAS
AL 30D BCAS
IF 30D BCAS
Pro-MMP-9

Figure 5E

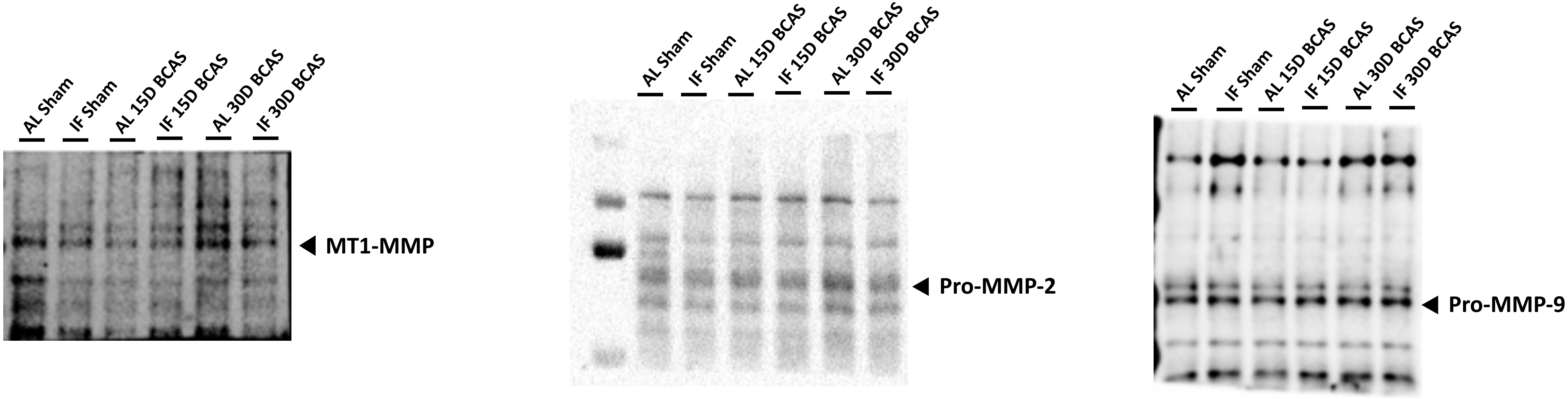
AL Sham
IF Sham
AL 15D BCAS
IF 15D BCAS
AL 30D BCAS
IF 30D BCAS
MT1-MMP
AL Sham
IF Sham
AL 15D BCAS
IF 15D BCAS
AL 30D BCAS
IF 30D BCAS
Pro-MMP-2
AL Sham
IF Sham
AL 15D BCAS
IF 15D BCAS
AL 30D BCAS
IF 30D BCAS
Pro-MMP-9

Figure 5G

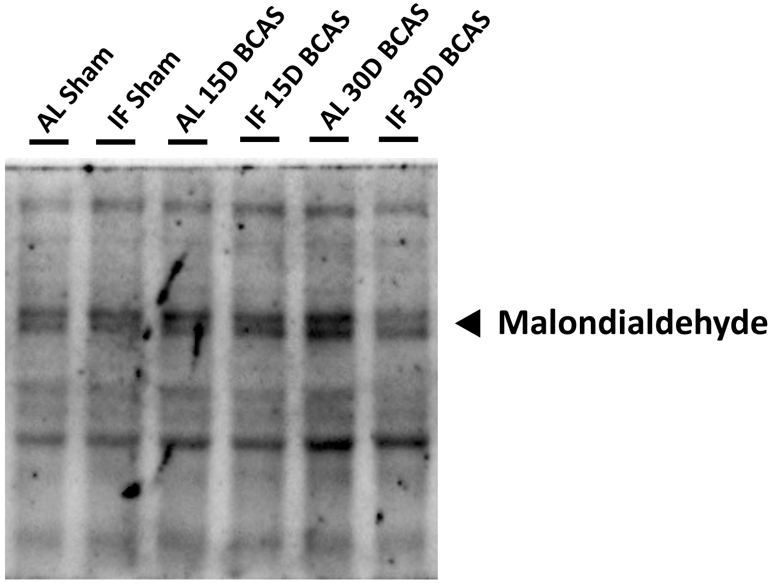

AL Sham
IF Sham
AL 15D BCAS
IF 15D BCAS
AL 30D BCAS
IF 30D BCAS
Malondialdehyde

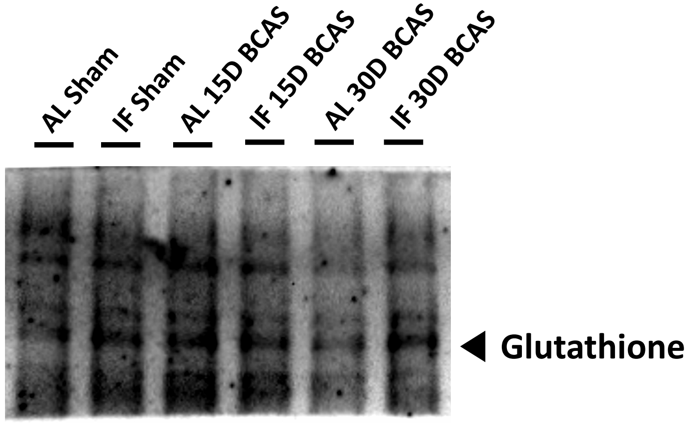

AL Sham
IF Sham
AL 15D BCAS
IF 15D BCAS
AL 30D BCAS
IF 30D BCAS
Glutathione

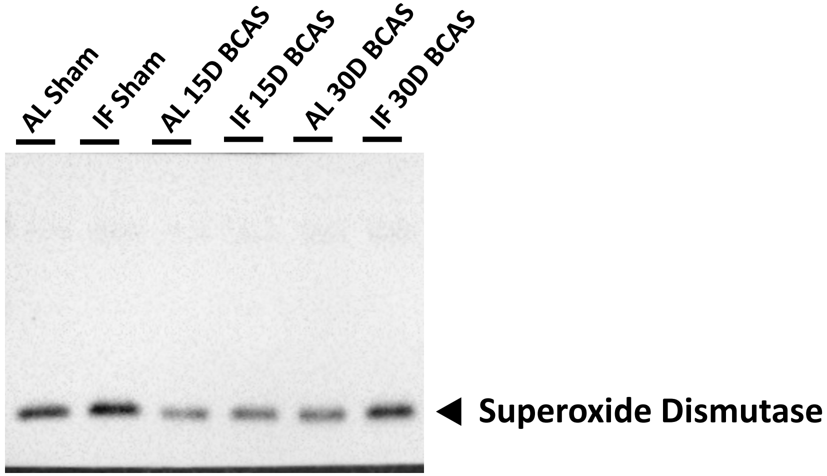

AL Sham
IF Sham
AL 15D BCAS
IF 15D BCAS
AL 30D BCAS
IF 30D BCAS
Superoxide Dismutase

Figure 5I

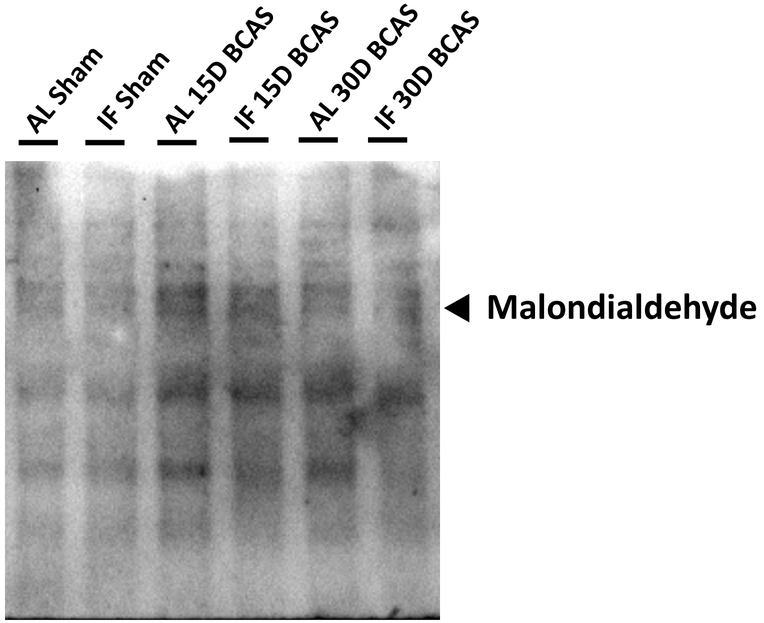
AL Sham
IF Sham
AL 15D BCAS
IF 15D BCAS
AL 30D BCAS
IF 30D BCAS
Malondialdehyde

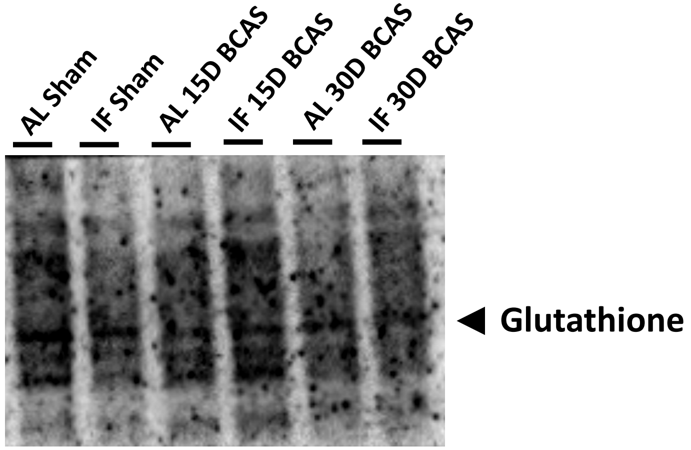
AL Sham
IF Sham
AL 15D BCAS
IF 15D BCAS
AL 30D BCAS
IF 30D BCAS
Glutathione

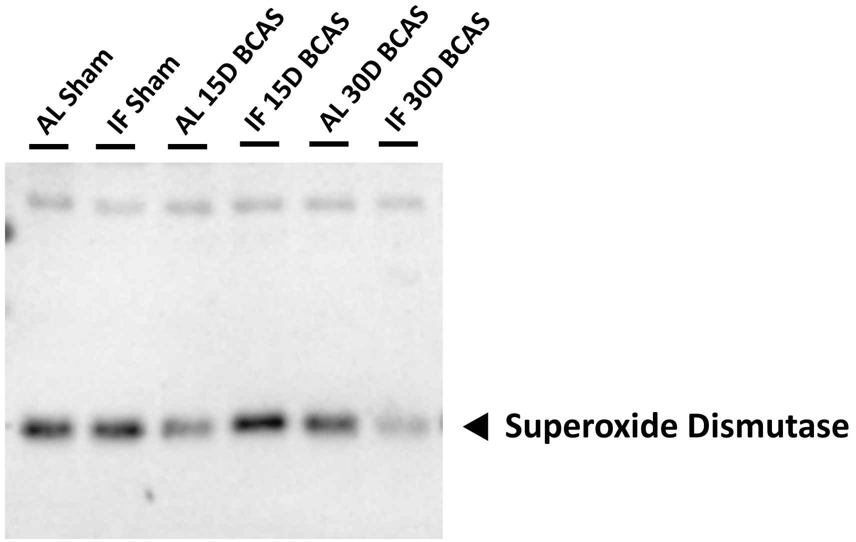
AL Sham
IF Sham
AL 15D BCAS
IF 15D BCAS
AL 30D BCAS
IF 30D BCAS
Superoxide Dismutase

Figure 5K

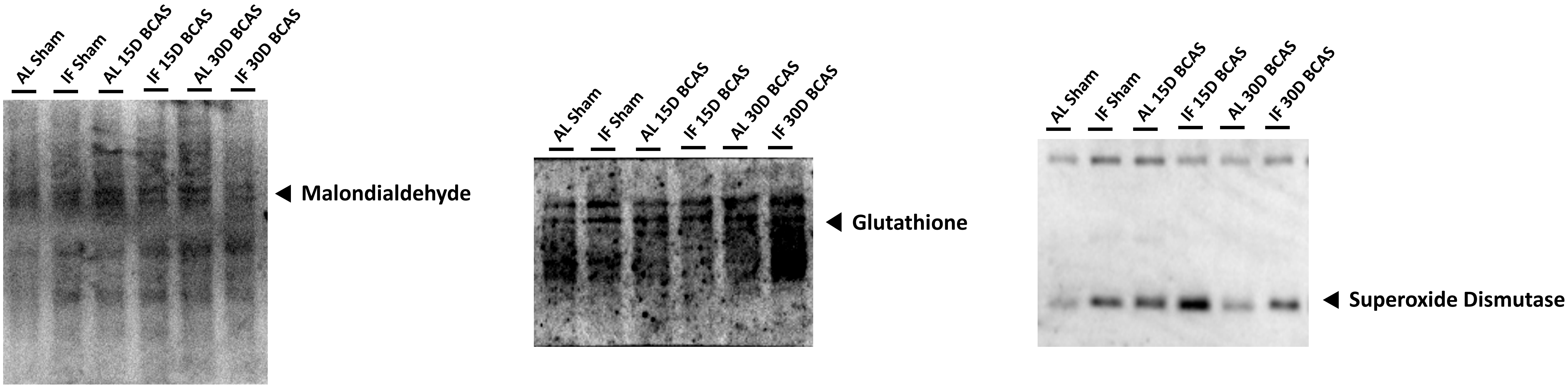
AL Sham
IF Sham
AL 15D BCAS
IF 15D BCAS
AL 30D BCAS
IF 30D BCAS
Malondialdehyde
AL Sham
IF Sham
AL 15D BCAS
IF 15D BCAS
AL 30D BCAS
IF 30D BCAS
Glutathione
AL Sham
IF Sham
AL 15D BCAS
IF 15D BCAS
AL 30D BCAS
IF 30D BCAS
Superoxide Dismutase
